# Systematic review of existing literature regarding the prevalence of pediatric atopic dermatitis in Honduras

**DOI:** 10.1016/j.jdin.2023.12.009

**Published:** 2024-01-06

**Authors:** Genevieve Patrick, Alexzandra Mattia, Julian Melchor, Madison Leonard, Ernest Quirindongo, Nicole Sangha, Brittany Long, Nicole Grant, Victoria Cruz, Anand “Sunny” Narayanan, Meihmy Chang, Charles Fleischer

**Affiliations:** a Florida State University College of Medicine, Tallahassee, Florida; b Florida State University, Tallahassee, Florida; c National Autonomous University of Honduras Medical College, Tegucigalpa, Honduras; d Universidad Católica de Honduras, San Pedro Sula, Honduras

**Keywords:** AD, atopic dermatitis, Central America, eczema, Honduras, Latin America, prevalence

## Abstract

**Background:**

Atopic dermatitis (AD) is an inflammatory skin condition, often multifactorial in origin, and most commonly manifests during childhood. Although there remains a deficit in literature, current data suggest Honduras may have the highest prevalence and severity of AD among all Latin American countries.

**Objective:**

To assess the current prevalence of pediatric AD in Honduras and evaluate existing gaps in available literature to monitor disease burden.

**Methods:**

A comprehensive literature search was performed in March 2023. Articles were removed if they were published before 2007, were of the incorrect study design, or were focused on countries outside of Honduras. The articles were independently reviewed by 2 authors.

**Results:**

The initial literature search yielded 174 studies, of which 7 met inclusion criteria. AD prevalence rates in children in Honduras ranged from 0.7% to 40.0%.

**Limitations:**

Limitations include elements of study design, analytic methods, study populations, and limited articles.

**Conclusion:**

There appears to be a disproportionately higher prevalence and disease burden of pediatric AD in Honduras. Future research should acquire accurate data to further understand the prevalence, incidence, and severity of AD in Honduras.

**Capsule Summary**

• We provide an update on the prevalence of atopic dermatitis in Honduras, which afflicts the most vulnerable patient populations.
• Addressing the deficit in literature describing atopic dermatitis in pediatric patients in Honduras may result in improved patient outcomes long-term.

## Introduction

Atopic dermatitis (AD), or eczema, is an inflammatory cutaneous condition often presenting on the scalp and flexor surfaces of the extremities with pruritic, erythematous papules or patches.^1^ Although AD ranks 15th among all nonfatal diseases in severity, it has the highest disease burden among cutaneous disorders as measured by disability-adjusted life-years.^1^ AD remains among the most prevalent skin conditions affecting adolescent populations (∼10%-20%), with a disproportionate occurrence in those from underdeveloped regions.^2^^,^^3^ Furthermore, previous studies suggest higher prevalence rates and more severe manifestations in Latin American countries.^3^, ^4^, ^5^, ^6^

The 1998 Phase 1 and 2002 to 2003 Phase 3 of the International Study of Asthma and Allergies in Childhood (ISAAC) study, a global cross-sectional questionnaire, demonstrated Honduras among the countries reporting the highest rates of self-reported symptoms suggestive of AD.^4^, ^5^, ^6^ Although the etiology for this variance remains ambiguous, these observations may be the result of an amalgamation of multiple variables, including genetic predisposition and environmental triggers.^2^

Despite significant worldwide prevalence, management of AD remains complex and multifaceted, with numerous treatment modalities available. Improved management may alleviate the negative effects of AD on quality of life, such as higher costs of medical care, sleep deprivation, absence from work or school, and declines in emotional and/or physical well-being of the patient and their caregiver.^2^^,^^3^ Considering the potential to mitigate the physical and psychological burdens of AD on Latin American communities through feasible and sustainable interventions, a thorough understanding of its prevalence and severity within this region is needed. The purpose of this review is to assess existing literature on the prevalence of pediatric AD in Honduras and identify gaps to be addressed in future studies.

## Methods

### Protocol adherence

This review adhered to the Preferred Reporting Items for Systematic Reviews and Meta-Analysis checklist. This review was not registered.

### Eligibility criteria

Published literature regarding the prevalence of AD among Latin American countries was included from the past 16 years (2007-2023). Sources were excluded if they met the following criteria: (1) were published before 2007, (2) focused on a region outside of Honduras, (3) focused on topics other than AD, (4) did not discuss the prevalence of AD, (5) were not cross-sectional analyses, and (6) were duplicates.

### Information sources and search strategy

PubMed, Cochrane, Embase, Medline (Ovid), GREAT, LILACS, Thesis, CDOPS, CRIDTC, COPECO, DESAS, BIMENA, and ADOLHN were utilized to identify sources adhering to the eligibility criteria in March 2023. Keywords used included “atopic dermatitis,” “eczema,” “atopic eczema,” “Honduras,” “Central America,” “Latin America,” “prevalence,” “incidence,” and “severity.” Additional data pertaining to AD prevalence were acquired from the Director of the Dermatology Residency Program and the Institutional Review Board Coordinator at the Universidad Nacional Autónoma de Honduras.

### Data selection and collection

Eligible articles were imported into Covidence, a systematic review management software, which automatically screened for duplicates. Two authors (AM and GP) independently screened titles and abstracts of the studies, excluding irrelevant sources. Following initial screening, AM and GP independently screened full-text articles, examining their adherence to eligibility criteria.

### Data synthesis

Data extraction was completed by 1 of 4 authors (GP, AM, NS, and BL). Consensus for article inclusion was completed by 2 authors (NS and AM), with a third author (GP) serving as an independent mediator to verify for accuracy and completion. The last name of the first author, publication year, country, study population characteristics, and prevalence of AD among both children and adults (if available) were extracted from each source.

### Risk of bias assessment

The Joanna Briggs Institute critical appraisal tool for case reports was used to determine the extent to which a case report has addressed the possibility of bias. Articles were reviewed for potential bias independently by 1 of the 4 reviewers.

## Results

### Study selection

**Fig 1:**
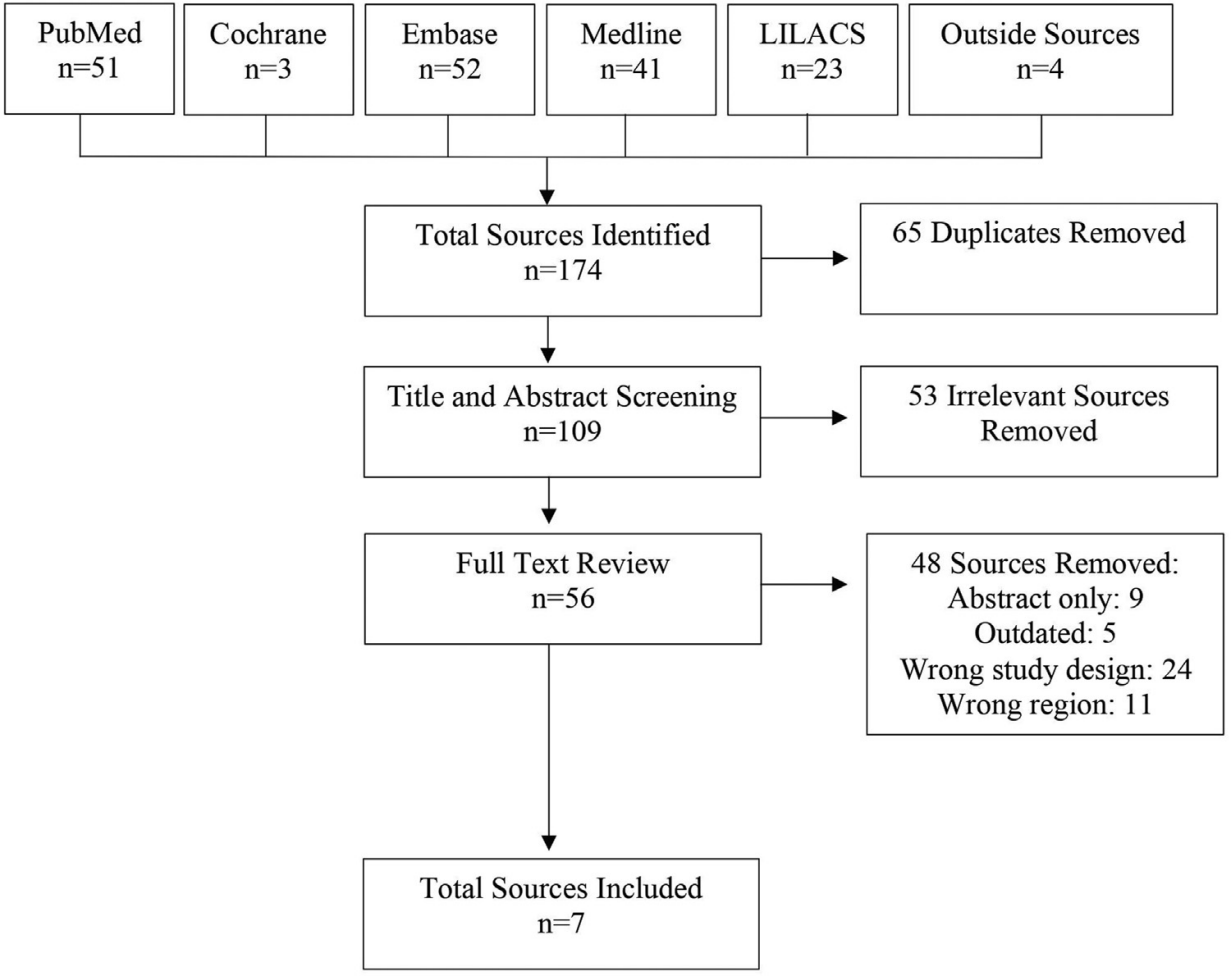
Preferred Reporting Items for Systematic Reviews and Meta-Analysis flowchart.

A total of 170 articles were identified across 13 databases: PubMed (*n* = 51), Cochrane (*n* = 3), Embase (*n* = 52), Medline (Ovid) (*n* = 41), GREAT (*n* = 0), LILACS (*n* = 23), Thesis (*n* = 0), CDOPS (*n* = 0), CRIDTC (*n* = 0), COPECO (*n* = 0), DESAS (*n* = 0), BIMENA (*n* = 0), and ADOLHN (*n* = 0). Four additional sources obtained from Universidad Nacional Autónoma de Honduras were added for screening. Following adherence to Preferred Reporting Items for Systematic Reviews and Meta-Analysis guidelines, as detailed in Fig 1, 7 eligible sources were included in this study.

### Summary of study characteristics

The 7 included cross-sectional studies pertaining to prevalence rates of AD among pediatric populations in Honduras were published between 2009 and 2019, although they may have utilized data predating publication (Table I). Only one source included participants over the age of 15 years.^7^ Two sources cited data extracted from the 2002 to 2003 ISAAC Phase 3 study.^8^^,^^9^ One source utilized data from the 2005 to 2007 International Study of Wheezing in Infants.^10^^,^^11^ Of the original research articles, 1 study was multicenter (Hernández et al^7^), including both rural and urban sites, whereas the other 2 were single center studies (Elvir-Mayorquin et al^12^ and Euceda et al^13^).

**Table I:** Summary of 7 studies demonstrating pediatric atopic dermatitis prevalence rates in Honduras

First author, year of data collection	Country(ies) of origin	Mean response rate (%)	Study population (*n*)	Prevalence of AD = % frequency (*n*)
Original research				
Hernández^7^, 2014	Honduras (227 sites, rural and urban)	N/A	Ages, <7 to >15 y: *n* = 15,002	Overall AD prevalence: 0.70% (*n* = 109) Pityriasis alba prevalence: 26.9% (*n* = 1316)
Elvir-Mayorquin^12^, 2015	Santa Rosa de Aguan, Department of Colón, Honduras	N/A	Ages, 6-15 y: *n* = 60	Overall AD prevalence: N/A Rhinitis: 30.0% (*n* = 18) Infantile asthma: 13.3% (*n* = 8) Family history of atopy: 50.0% (*n* = 9)
Euceda^13^, 2015	Northwestern Regional Hospital-Honduran Social Security Institute (IHSS-HRN) in Honduras	65.9%	Ages, 0 mo to 11 y: *n* = 329	Overall AD prevalence: 40.0% (*n* = 133) AD prevalence in infants: 62.5% (*n* = 83) AD prevalence in preschoolers: 36.0% (*n* = 48) AD prevalence in schoolchildren: 1.5% (*n* = 2)
Expansion of ISAAC				
Odhiambo^8^, 2002-2003∗	Ages, 6-7 y: 60 countries	86.2%	Ages, 6-7 y: *n* = 1907	Current symptoms: 15.9% (*n* = 304) Current severe symptoms: 3.9% (*n* = 74) Lifetime AD prevalence: 13.6% (*n* = 260)
	Ages, 13-14 y: 96 countries	91.4%	Ages, 13-14 y: *n* = 2675	Current symptoms: 15.6% (*n* = 416) Current severe symptoms: 3.6% (*n* = 95) Lifetime AD prevalence: 7.7% (*n* = 207)
Solé^9^, 2002-2003∗	Ages, 6-7 y: 14 Latin American countries	86.2%	Ages, 6-7 y: *n* = 1907	Current symptoms: 15.9% (*n* = 304) Current severe symptoms: 3.9% (*n* = 74) Lifetime AD prevalence: 13.6% (*n* = 260)
	Ages, 13-14 y: 17 Latin American countries	91.4%	Ages, 13-14 y: *n* = 2675	Current symptoms: 15.6% (*n* = 416) Current severe symptoms: 3.6% (*n* = 95) Lifetime AD prevalence: 7.7% (*n* = 207)
Draaisma^10^, 2005-2007†	4 European and Central American countries, including San Pedro Sula, Honduras	85%	Ages, 0 mo to 1 y: *n* = 779	AD in first year of life: 28.2% (*n* ≈ 220) Family history of AD: 21.4% (*n* ≈167) Family history of asthma: 23.5% (*n* = 183) Family history of rhinitis: 28.9% (*n* ≈ 225)
Folgar^14^, 2016	3 regions in Honduras (Comayagua, Santa Rosa de Copán and Cuyalí, and El Paraíso)	N/A	Ages, 6-7 y: *n* = 283	Overall AD prevalence: 33.2% (*n* = 94)
			Ages, 13-14 y: *n* = 339	Overall AD prevalence: 20.9% (*n* = 71)

The symbol (≈) denotes estimations of (*n*) based on percentages reported.

*AD*, Atopic dermatitis; *ISAAC*, International Study of Asthma and Allergies in Childhood; *N/A*, not available.

∗ Data depicted in chart are obtained from single study site in San Pedro Sula, Honduras in ISAAC Phase 3 study (2002-2003).

† Data depicted in chart are obtained from expansions of International Study of Wheezing Infants in one center in San Pedro Sula, Honduras.

### Summary of results of individual studies

#### Original research

Between 2014 and 2015, 3 original studies were conducted in Honduras.^7^^,^^12^^,^^13^ In 2014, Hernández et al^7^ sampled 15,002 students from 227 sites, both rural and urban, to assess the prevalence of pediatric dermatologic conditions. They reported an overall AD prevalence rate of 0.7% (*n* = 109) with significant associations with rhinitis and asthma in addition to an increased risk of AD co-occurrence at >3.3 times and 3.6 times, respectively (*P* < .01).^7^ Similarly, a family history of either condition resulted in an increased risk of AD (2.4× for rhinitis, 1.7× for asthma, *P* < .01). Furthermore, the second most common dermatosis observed in this study (8.8%, *n* = 1320) was pityriasis alba, a postinflammatory hypopigmentation disorder often associated with AD. A statistically significant association was identified between pityriasis alba and outdoor education time during the hours of greatest UV exposure (between 9:00 am and 3:00 pm) (*P* < .05).^7^ Elvir-Mayorquin et al^12^ similarly analyzed rates of dermatoses in 60 Honduran school children of Afro-descent and found a 20% prevalence of pityriasis alba, however, they reported no incidence of active AD among the study population. Euceda et al^13^ conducted a cross-sectional study among 329 children (ages, 0 months to 11 years) sampled from a pediatric dermatological outpatient clinic in the northern part of Honduras and found an overall AD prevalence of 40% (*n* = 133). Of those affected by AD, 62.5% (*n* = 82) cases occurred in infants

#### Expansion of ISAAC

In 2009, Odhiambo et al^8^ published results from an expansion of ISAAC Phase 1 that included additional data from over 100 new centers. Data obtained in 2002 from a clinical site located in San Pedro Sula demonstrated that current prevalence rates for active eczema symptoms were 15.9% and 15.6% for 6- to 7-year-olds and 13- to 14-year-olds, respectively. Lifetime prevalence rates for AD, however, were reported at 13.6% and 7.7% for 6- to 7-year-olds and 13- to 14-year-olds, respectively.^8^ Similarly, Solé et al^9^ evaluated prevalence rates of eczema at centers participating in ISAAC Phase 3 in the 6- to 7-year-old and 13- to 14-year-old age groups.^9^ Data obtained from San Pedro Sula, Honduras, reflected prevalence rates of 13.6%, 15.9%, and 3.9% for 6- to 7-year-olds and 7.7%, 15.6%, and 3.6% for 13- to 14-year-olds experiencing eczema ever in their lifetime, experiencing current symptoms of eczema, and those experiencing current severe eczema symptoms, respectively.^9^ The prevalence of eczema in all categories was negatively associated with the latitude of the center. Similarly, a significant inverse relationship existed between the prevalence of sleep disturbance due to AD and the latitude of the center.

From 2005 to 2007, Draaisma et al^10^ conducted an extension of ISAAC with additional study design modifications utilizing data from the 2005 to 2007 International Study of Wheezing in Infants to specifically assess the international prevalence and risk factors of wheezing in infants as well as the presence of pediatric AD and any associated risk or protective factors.^11^ Data were collected from 779 infants (85% response rate) and demonstrated a 28.2% prevalence of AD within the first year of life. Furthermore, an overall AD prevalence rate of 18.2% was found for study sites located in Central America, which was significantly higher than the European prevalence at 14.9% (*P* < .001). Meta-analysis of associated factors demonstrated significant relationships between large family size and the development of AD (*P* < .05). Conversely, higher maternal education status and breastfeeding for at least 3 months were associated with a decreased risk of developing AD (*P* < .05).^10^

In 2016, Folgar et al^14^ expanded ISAAC Phase 1 to include 622 adolescents located across 3 regions (Comayagua, Santa Rosa de Copán and Cuyalí, and El Paraíso). An AD prevalence rate of 26.5% was reported, with higher prevalence rates associated with men and individuals aged 6 to 7 years.^14^

### Bias and confidence assessment

A cross-sectional study design was a major limitation among several studies.^7^, ^8^, ^9^, ^10^, ^11^, ^12^, ^13^, ^14^ Odhiambo et al^8^ reported limited study coverage in the younger age group and in some regions with participating urban centers located primarily along coastal areas, leading to poor generalizability. Additionally, pruritic conditions may confound overestimated prevalence rates of AD, particularly in low-resource settings. Likewise, Euceda et al^13^ reported participants were primarily from a highly tropical northern zone. Hernández et al^7^ reported limitations in study enrollment for some schools located in rural regions, requiring the inclusion of students outside of the third to sixth grade target range. Elvir-Mayorquin et al^12^ had limited study enrollment from a specific patient population from one study site located in the northern region of Honduras.

Concerns regarding generalizability of the survey results was another theme identified throughout multiple sources. Draaisma et al^10^ stated the survey utilized in their study was not specifically geared toward AD and had an inadequate sample size, affecting the validity of responses. Solé et al^9^ cautioned against the correlation of eczema symptoms and socioeconomic parameters, as individual center data may differ from national data. Folgar et al^14^ commented on possible bias regarding Phase 1 of the ISAAC study design wherein clinical assessment with diagnosis based on patient history was lacking, and questionnaires were filled out by parents of those aged between 6 and 7 years. Furthermore, the 3 study regions share similar climatic features of high humid, temperate weathers which may overestimate the generalizability of the study’s findings.^14^

## Discussion

An expanded literature review including both cross-sectional and prevalence studies assessing AD prevalence among pediatric populations from neighboring countries in the Central and South American region, such as Colombia, Bolivia, Guatemala, Costa Rica, Ecuador, Argentina, Brazil, and Mexico, were reviewed for comparison to Honduran population observations.^15^, ^16^, ^17^, ^18^, ^19^, ^20^, ^21^, ^22^, ^23^ Generally, overarching trends included the increased prevalence rates of AD among tropical regions, increased severity of AD manifestations among affected individuals within the study’s scope of region, and an increased likelihood of developing AD for those with a positive family history. The results of this review support hypotheses surrounding the global increase in the prevalence rates of AD among pediatric populations, with particular attention to the incremental escalation of rates in Latin American countries. Since ISAAC Phase 1 in 1999, expansions of the study and novel research endeavors have highlighted increased overall prevalence and severity of AD among individuals in Honduras.^4^^,^^7^^,^^12^^,^^13^

Subsequent phase studies demonstrated that 44 out of 52 centers participating in Phase 1 of ISAAC, including Honduras, observed an increase in the overall prevalence rates of AD among the 6- to 7-year-old group, whereas 47 of 79 sites reported increases in AD rates for children aged 3 to 4 years.^1^ According to Phase 3 of ISAAC in 2010, the prevalence of AD among pediatric populations in Honduras was among the highest observed.^6^ Furthermore, Honduras ranked third out of 65 countries for prevalence of current severe AD symptoms among participants aged 6 to 7 years and 10th out of 98 countries for participants aged 13 to 14 years.^8^ Despite availability of recent data, Honduras appears to have significant burden of disease with an increased incidence of severe manifestations across both ISAAC age groups comparatively despite limited recent data.^9^ The variance in climate observed regionally and even nationally may contribute to the localization of these findings.^14^

Although successful in producing global AD prevalence data, the limitations of ISAAC have been perpetuated through subsequent studies. For example, there remain insufficient available data regarding AD in Honduran adult populations despite evidence from neighboring countries suggesting increased prevalence. Moreover, Honduran prevalence rates of AD were primarily based on data from one clinical center in San Pedro Sula, which exclude a majority of the country’s population.^8^^,^^9^ Limited age ranges and restricted geographical distribution of ISAAC participants may lead to misrepresentation of true overall prevalence rates.^10^^,^^21^ Furthermore, although the ISAAC analysis was centered around the completion of self-reported questionnaires utilizing the Hanifin and Rajka criteria (10%-100% sensitivity and 89%-99% specificity), physician-diagnosed AD was consistently lower than self-reported AD prevalence.^1^^,^^21^ These findings may be related to the study’s inclusion of individuals without clinically diagnosed eczema as well as potentially vague manifestations and presentations that widely vary among skin types. Finally, the translational adaptations of the surveys may have decreased the validity of the ISAAC questionnaire and overestimated prevalence rates.^8^, ^9^, ^10^^,^^12^^,^^21^

Despite the high prevalence of reported symptoms, 59% of children aged 5 to 6 years lacked a formal diagnosis or treatment plan.^16^ Considering the significant impacts on overall health and quality of life, an opportunity presents to obtain a more thorough understanding of the diagnostic and treatment barriers existing for Honduran individuals.^23^ Cultural, socioeconomic, and transportation barriers may prohibit individuals from obtaining necessary medical intervention. Our study contributes to further understanding these limitations, which may allow enhanced management, mitigation of disease burden, and improved quality of life.

## Conclusion

Future research should focus on acquiring data relevant to understanding the prevalence, incidence, and severity of AD among pediatric and adult populations throughout Honduras utilizing standardized surveys reporting to a centralized data repository. Finally, partnerships with local health care providers and the provision of educational materials may increase the validity of AD diagnosis and improve treatment adherence.

## Conflicts of interest

None disclosed.
